# Supplementary figures and images for: Genetic admixture patterns in Argentinian Patagonia
Source: PLoS One. 2019 Jun 17;14(6):e0214830. doi: 10.1371/journal.pone.0214830 (PMC6576754; doi:10.1371/journal.pone.0214830)

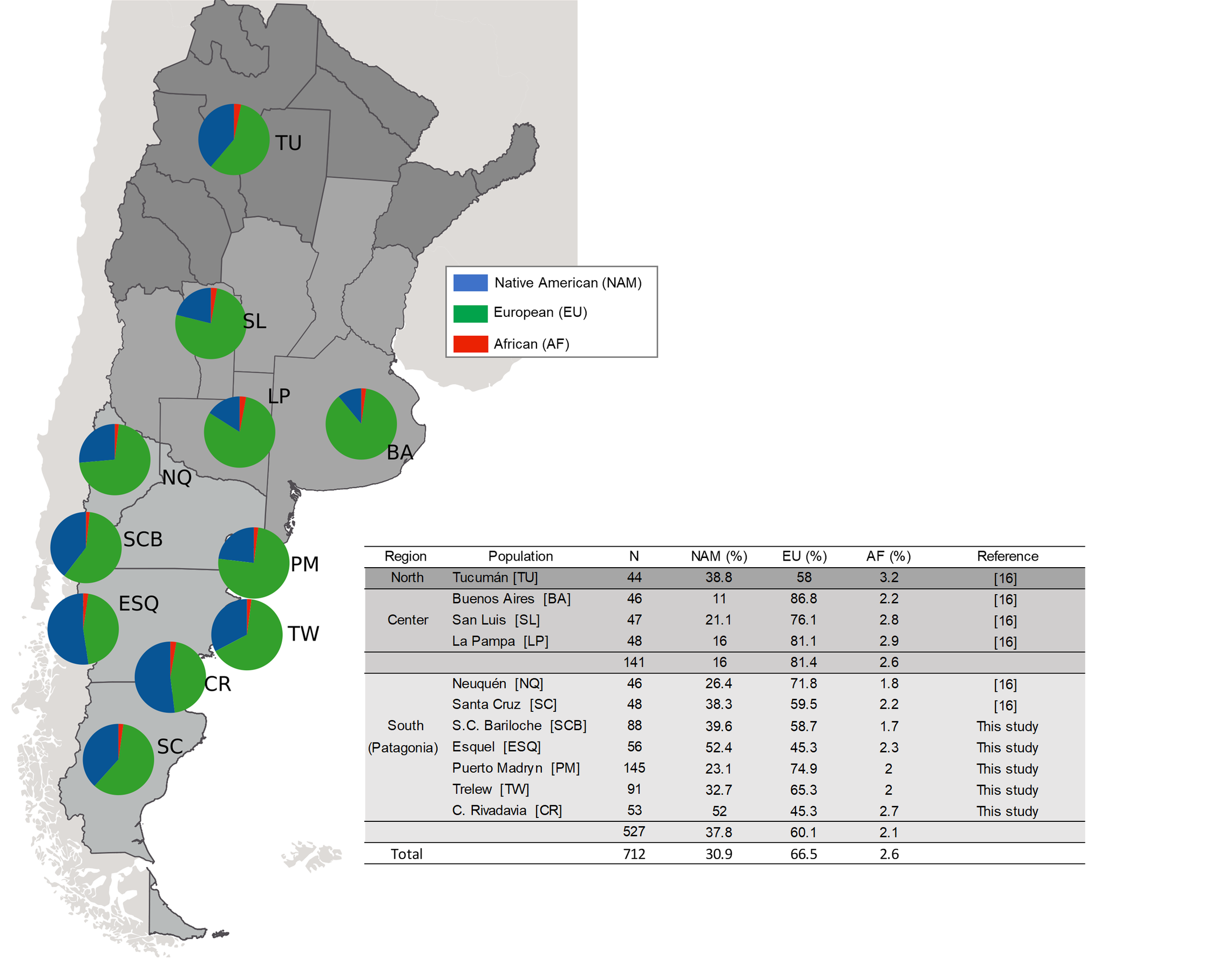

Supplement: S1 Fig — (TIF) [file pone.0214830.s001.tif]
